# Comparison of the ImmuView and the BinaxNOW antigen tests in detection of *Streptococcus pneumoniae* and *Legionella pneumophila* in urine

**DOI:** 10.1007/s10096-017-3016-6

**Published:** 2017-06-06

**Authors:** S. Athlin, A. Iversen, V. Özenci

**Affiliations:** 1 Department of Infectious Diseases, Faculty of Medicin and Health, Örebro University, S-701 85 Örebro, Sweden; 2 Division of Clinical Microbiology, Department of Laboratory Medicine, Karolinska Institutet, Stockholm, Sweden; 3 Department of Clinical Microbiology, Karolinska University Hospital, Solna, Stockholm Sweden; 4 Department of Clinical Microbiology F 72, Karolinska Institutet, Karolinska University Hospital, SE 141 86 Huddinge, Stockholm Sweden

## Abstract

The use of urinary antigen tests (UATs) may provide early etiology in pneumonia, and facilitates rapid and directed antibiotic treatment. In this study, we evaluated the novel lateral flow ImmuView *Streptococcus pneumoniae* and *Legionella pneumophila* UAT, which detects pneumococcal and *L. pneumophila* serogroup 1 antigens in a combined test. We compared the ImmuView UAT with the BinaxNOW *S. pneumoniae* UAT and the BinaxNOW *L. pneumophila* UAT in 147 patients with pneumococcal bacteremia (*n* = 48), non-pneumococcal non-*Legionella* bacteremia (*n* = 93) and *Legionella* infections in the lower airways (*L. pneumophila*, *n* = 5; *L. bozemanii*, *n* = 1). In three cases, the ImmuView test was invalid before and after boiling while the BinaxNOW tests were valid in all cases. In 144 cases, the three UATs demonstrated a very good inter-assay agreement for detection of pneumococcal antigen (*κ* = 0.86) and *L. pneumophila* antigen (*κ* = 1.00). The ImmuView and BinaxNOW *S. pneumoniae* tests had similar sensitivities (62% vs 60%; *p* = ns) in 48 cases with pneumococcal bacteremia and both tests had specificities of 97% in 96 cases with non-pneumococcal infections. Furthermore, the ImmuView and BinaxNOW *L. pneumophila* tests were positive for *Legionella* antigen in five patients with confirmed *L. pneumophila* serogroup 1 infections, and negative in all non-*L. pneumophila* cases. The ImmuView and BinaxNOW tests performed similarly when evaluated on urine samples from bacteremic and non-bacteremic patients with identified etiology.

## Introduction

The use of urinary antigen tests (UATs) is recommended in hospitalized patients with community-acquired pneumonia to support early and directed antibiotic therapy [1, 2]. Antigen detection in urine has many advantages, since urine is excreted in large quantities and collection does not require invasive methods [3]. In previous studies, a positive UAT result was supportive for the clinician to narrow the antibiotic treatment [4–6]. However, in a recent study by Falguera et al., detection of *Streptococcus pneumoniae* and *Legionella pneumophila* antigens by UATs did not associate with improved patient outcome or cost-effectiveness [7]. By the development of novel UATs, with enhanced performance of detecting these pathogens, the clinical and economic benefits by using UATs may be improved.

Several monovalent UATs are available for detection of *S. pneumoniae* and *L. pneumophila* polysaccharides by separate tests [3, 8, 9]. Recently, the combined lateral flow test, the ImmuView® *S. pneumoniae* and *L. pneumophila* (Statens Serum Institut Diagnostica (SSID), Denmark) UAT was introduced on the market. The test is based on immunochromatographic technology for detection of *S. pneumoniae* and *L. pneumophila* serogroup 1 antigens and is the first UAT that detects both pathogens in a single test.

According to Jørgensen, Uldum (both Statens Serum Institut, Denmark) and co-authors (SSID), the combined sensitivity of the ImmuView test for detection of *S. pneumoniae* and *L. pneumophila* antigens was higher than of three competing tests in combination (87% vs 74%) [10]. The specificity of the test showed high rates (100% for *L. pneumophila* serogroup 1 and 99% for *S. pneumoniae*) but did not include bacteremic patients with non-*S. pneumoniae* non-*Legionella* etiology. False-positive UAT results have been observed in patients with positive blood cultures (BC) for α-hemolytic streptococci and Gram-negative bacteria previously [11, 12]. Therefore, the analyses of both sensitivity and specificity have utmost clinical relevance for targeting antibiotic treatment according to UAT results. In this study, we aimed to evaluate the performance of the ImmuView test on bacteremic patients with identified pneumococcal and non-pneumococcal etiology, as well as on patients with Legionnaires’ disease.

## Materials and methods

Urine samples from adult patients (≥18 years) with community-acquired infections were collected between September 2013 and October 2016 at Örebro University Hospital, Karolinska University Hospital Huddinge, and Skåne University Hospital Malmö. Inclusion criteria were urine samples from (i) bacteremic patients with simultaneous blood culture positivity or (ii) non-bacteremic patients with lower respiratory tract samples containing *Legionella* spp. Culture on blood and urine was performed by standard methods. One sample per patient was analyzed. Cases with coagulase-negative staphylococci in only one BC bottle were excluded. Isolated bacteria from BC were identified to species level according to standard methods. *L. pneumophila* cases were identified by PCR on BAL or sputum and confirmed by Binax® *L. pneumophila* serogroup 1 enzyme immunoassay (EIA; Alere, USA) on urine. One *L. bozemanii* case was identified by culture and PCR for *Legionella* spp. on BAL and sputum and the isolate was determined on species level by matrix-assisted laser desorption ionization-time of flight mass spectrometry (MALDI Biotyper®; Bruker Daltonics, USA). All clinical samples, along with standard microbiological test results, age and gender were anonymized before storage at −20 °C.

All urine samples were thawed in room temperature for a blinded and simultaneous testing procedure with the ImmuView *S. pneumoniae* and *L. pneumophila* test, the BinaxNOW *S. pneumoniae* test, and the BinaxNOW *L. pneumophila* test. The ImmuView test is a combined lateral flow test based on immunochromatographic technology with one control line for validation and two test lines for detection of *S. pneumoniae* and *L. pneumophila* serogroup1 antigens, respectively. The test was performed according to the manufacturer’s instructions, as three drops of urine and two drops of running buffer were added to a test tube and gently whirled. The test strip was then inserted into the tube, and test results were read after 15 min. Any visible pink/red (*S. pneumoniae*) or blue (*L. pneumophila*) test line was considered as a positive result. A test result was considered invalid if the control line was absent or if the test showed three gray/purple test lines. The samples were vortexed for 5 s but not boiled or concentrated before the first testing procedure. Any urine sample with discrepant or invalid test result was re-tested, as well as all urine samples with a positive result for *L. pneumophila* serogroup 1. The re-testing procedure was done before boiling and after boiling for 10 min. Invalid tests were examined regarding levels of leucocytes, erythrocytes, and glucose levels by Multistix® 7 (Siemens Healthcare Diagnostics, USA).

The inter-assay agreement between the tests was estimated by calculating Cohen’s unweighted kappa coefficient (*κ*). McNemar’s test was used for the comparison of sensitivity and specificity rates. A confidence interval (CI) of 95% was used for statistical precision. A two-tailed *p*-value of <0.05 was considered statistically significant. The statistical analyses were performed with a statistical software package (SPSS for Windows, version 17.0).

## Results

Of 147 cases included in this study, 141 were bacteremic cases (median age, 74 years (range, 18–96 years); female, 44%) with isolated bacteria from BC as shown in Table 1. In addition, six non-bacteremic cases with lower respiratory tract samples containing *L. pneumophila* serogroup 1 [*n* = 5; median age, 57 years (range, 52–82 years); all males] and *Legionella bozemanii* (*n* = 1; age, 42 years; male) were included.

**Table 1:** Blood culture isolates of 141 bacteremic patients in the study

Blood culture isolate	No. of patients
*Streptococcus pneumoniae*	48
*Escherichia coli*	28
*Staphylococcus aureus*	12
*Enterococcus faecalis*	8
*Staphylococcus epidermidis*	5
*Klebsiella pneumoniae*	4
*Proteus mirabilis*	4
*Pseudomonas aeruginosa*	3
*Streptococcus mitis*	3
*Klebsiella oxytoca*	2
Other*	24

*One each of *Bacillus cereus, Bacteroides aerogenes, Bacteroides caccae, Bacteroides fragilis, Clostridium paraputrificum, Enterococcus cloacae, Granulicatella adiacens*, Group G streptococcus, *Listeria monocytogenes, Morganella morganii, Salmonella typhimur, Streptococcus agalactiae, Streptococcus anginosus, Streptococcus bovis, Streptococcus pyogenes, Streptococcus salivarius, Clostridium perfringens + E. coli, E. faecalis* + α-hemolytic streptococcus, *E. faecalis* + *P. mirabilis, E. faecalis* + *S. agalactiae, E. faecium* + *P. aeruginosa, C. perfringens* + *E. faecium* + *S. epidermidis, C. perfringens* + *K. pneumoniae* + *S. anginosus* and *S. epidermidis* + *S. salivarius* + *S. mitis*

Table 2 shows the test results of the three UATs obtained by category of infection. In three cases (2.1%), the ImmuView tests were invalid since no control lines were observed. In one case (*E. coli* in BC), the test line for pneumococcal antigen turned gray, and no other lines were visible at the initial testing as well as after boiling. In the second case (*E. coli* in BC), the test line for pneumococcal antigen turned gray, and no other lines were visible but all three lines turned gray after boiling. In the third case (*P. mirabilis* in BC), no lines were visible at the initial testing or after boiling. In the first case, the urine sample contained a high level of glucose (28 mmol/l) and all three cases showed elevated levels of leucocytes (>70 leucocytes/μl) and erythrocytes (>80 erythrocytes/μl). The third sample was highly viscous before as well as after boiling. In these three samples, the BinaxNOW tests were valid and the results were negative.

**Table 2:** Test results of the ImmuView *S. pneumoniae* and *L. pneumophila* UAT in comparison with the BinaxNOW *S. pneumoniae* UAT and the BinaxNOW *L. pneumophila* UATs in 147 bacteremic and non-bacteremic patients

		ImmuView UAT		BinaxNOW UATs	
Category of infection	No. tested (*n* = 147)	No. positive (%) *S. pneumoniae*	No. positive *L. pneumophila*	No. positive (%) S*. pneumoniae*	No. positive *L. pneumophila*
Bacteremic, pneumococcal	48	30/48^a^ (62)	0	29/48^a^ (60)	0
Bacteremic, non-pneumococcal	93	3/93^b^ (3)	0	3/93 (3)	0
Non-bacteremic *L. pneumophila*	5	0	5/5	0	5/5
Non-bacteremic *L. bozemanii*	1	0	0	0	0

*UAT* urinary antigen test

^a^In 26 of 48 pneumococcal bacteremia cases, the ImmuView and the BinaxNOW *S. pneumoniae* UATs showed concordant positive test results. In seven cases, the UATs showed discrepant results

^b^The ImmuView UAT showed valid test results in 90 of 93 non-pneumococcal bacteremia cases, three of which showed positive test results

Calculated on 144 of 147 cases (98%) with valid test results, the inter-assay agreement between the ImmuView test and the BinaxNOW *S. pneumoniae* test for detection of pneumococcal antigen was very good (*κ* = 0.86; CI, 0.76–0.96). Both tests were positive in 29 cases and negative in 108 cases, and with discrepant results in seven cases of pneumococcal bacteremia (Table 2). Discrepant results were confirmed by re-testing. The inter-assay agreement between the ImmuView test and the BinaxNOW *L. pneumophila* test for detection of *L. pneumophila* serogroup 1 antigen was very good (*κ* = 1.00; CI, 1.00–1.00); both tests were positive in five cases and negative in 139 cases.

The performance rates were calculated on cases with valid test results for all three tests. For detection of pneumococcal antigen in urine, the sensitivity in patients with pneumococcal bacteremia was 62% (30/48) for the ImmuView test and 60% (29/48; *p* = ns) for the BinaxNOW *S. pneumoniae* test. Calculated on non-pneumococcal cases (bacteremic, *n* = 90; non-bacteremic *Legionella* spp., *n* = 6), both tests yielded specificity rates of 97% (93/96), since both tests showed false-positive results in three cases with non-pneumococcal bacteremia (*E. coli*, *E. faecalis* and *S. epidermidis*). The false-positive test results were confirmed by re-testing the samples.

The ImmuView and the BinaxNOW *L. pneumophila* tests were positive for *Legionella* antigen in all cases (5/5, 100%) with *L. pneumophila* serogroup 1 etiology, and the tests were positive at re-testing before and after boiling for 10 min. Furthermore, both tests were negative in all cases (139/139; 100%) with non-*L. pneumophila* etiology, including the *L. bozemanii* case.

## Discussion

The ImmuView *S. pneumoniae* and *L. pneumophila* UAT may provide benefits in terms of reduced laboratory work due to the detection of both pathogens in a single test. However, the combination of two assays is of concern, since the duality of the test may influence the test performance negatively. In this study, the ImmuView test showed a very good inter-assay agreement in comparison with the BinaxNOW *S. pneumoniae* UAT and the BinaxNOW *L. pneumophila* UAT in infected patients with identified etiology. Thus, our study showed that the ImmuView test performed similarly to the BinaxNOW tests in bacteremic and non-bacteremic patients.

For detection of pneumococcal antigen in bacteremic patients, the sensitivities were similar of the ImmuView and the BinaxNOW *S. pneumoniae* tests (62% vs 60%; *p* = ns). In comparison, sensitivities of the BinaxNOW test ranged between 63 and 88% in bacteremic patients in previous studies [9]. In the study by Jørgensen et al., the sensitivity was 84% for the ImmuView test and 78% for the BinaxNOW test (*p* = ns) on un-boiled urine samples from bacteremic patients [10]. In addition, the ImmuView was recently compared against the BinaxNOW *S. pneumoniae* test and two *L. pneumophila* EIAs, as described by Lindsay et al. [13]. The test was positive in 70/78 (valid test results, 90%) urine samples from previously *Legionella* positive patients and was positive for pneumococcal antigen in 45/554 (8%) patients with undefined status of infection.

Cross-reactivity between *S. pneumoniae* and other bacterial species may yield false-positive results in patients with non-pneumococcal infections. In the present study, the ImmuView and the BinaxNOW *S. pneumoniae* tests were both positive for pneumococcal antigens in three bacteremic cases with non-pneumococcal etiology, yielding specificities of 97% for both tests (Table 2). Similarly, Smith et al. found that the BinaxNOW test was positive in 3/106 adult patients with community-acquired septic infections caused by *E. coli*, *K. pneumoniae* and *Enterobacter* spp. in BC, yielding a specificity of 97% [11]. When Jørgensen et al. evaluated the specificity of the ImmuView test in 76 non-bacteremic patients with urinary tract infections, the test was negative for pneumococcal antigen in all but one case (specificity, 99%), while the BinaxNOW test was negative in all cases [10]. Furthermore, they noted that the test was positive for both pneumococcal and *Legionella* antigens in 4/71 (6%) cases with pneumococcal bacteremia, which was considered as either double infections or due to false-positivity [14]. Dual-positivity of the ImmuView test was also observed in 8/45 patients (18%) with unknown etiology in the study by Lindsay et al. [13], but in the present study no dual-positivity was observed.

Previously, when we evaluated the Uni-Gold *S. pneumoniae* UAT on bacteremic patients, we observed false-positive test results in 15% (7/48) of patients with *E. coli* and in 32% (6/19) of patients with α-hemolytic streptococcus etiology respectively [12]. The Uni-Gold test was developed by the same manufacturer as the ImmuView test (SSID, Denmark) and was based on similar lateral flow immunochromatographic technology for detection of pneumococcal antigen. However, in the present study, we noted false-positive results for the detection of pneumococcal antigen in only one case of 39 (3%) of *E. coli* bacteremia, and in no case of α-hemolytic streptococcus bacteremia (*n* = 9). This may indicate that the ImmuView and the Uni-Gold tests actually differ in specificity, presumably due to variations in test technologies.

For detection of *L. pneumophila* serogroup 1 antigen in urine, the ImmuView test and the BinaxNOW *L. pneumophila* test performed similarly, yielding positive results in all five *L. pneumophila* serogroup 1 cases and negative in all other cases, including one *L. bozemanii* case. Calculated on this limited set of samples, the sensitivity and the specificity rates yielded 100% for each test. In comparison, in a meta-analysis by Shimada et al., the pooled sensitivity and specificity rates for the BinaxNOW *L. pneumophila* test were 90% and 99% respectively [8]. Jørgensen et al. reported sensitivities and specificities in patients with urinary tract infections of 87% and 100% for the ImmuView test, and 78% and 100% for the BinaxNOW test respectively [10]. However, in 2/55 (4%) of confirmed *L. pneumophila* serogroup 1 cases, the test was negative for *Legionella* but positive for *S. pneumoniae*, which was interpreted as either double infection or false-positivity as described above.

We used PCR on lower respiratory samples for identification of *Legionella* cases, which is suggested to be a reliable method for the diagnosis of Legionnaires’ disease [15, 16]. PCR is more sensitive than airway culture and UAT for detection of *Legionella* spp., which may be accredited to a high detection rate at a low bacterial load [17, 18]. Moreover, we used the Binax *L. pneumophila* serogroup 1 EIA to determine the serogroup of PCR positive *L. pneumophila* cases. The sensitivity of this assay has been demonstrated to be moderate (74%), but with high specificity (99%), and was used as a reference method to evaluate the BinaxNOW *L. pneumophila* test previously [10, 19]. Accordingly, we consider the positive results by the ImmuView and BinaxNOW in our study to be reliable, as we used the combined reference standard of PCR and EIA. However, due to the low number of cases with *L. pneumophila* serogroup 1 etiology and the fact that the cases were not determined on a subgroup level, we may have overlooked potential differences in performance between the tests, and further analysis with larger group of patients are warranted.

In three cases, the ImmuView tests were invalid while the BinaxNOW tests were valid in all cases. High viscosity was the presumable reason for invalidity in the first case, but in the two other cases the cause of invalidity remains unknown. In the study by Lindsay et al., the ImmuView showed invalid test results when applied on 7/554 retrospectively collected and frozen urine samples (1%) [13]. In five cases, the samples derived from patients with previously positive culture, serology, or Binax EIA for *L. pneumoniae* but no other information of the samples or the test lines are available. In the study by Jørgensen et al., all urine samples were frozen at −20 °C, and showed valid test results after they had been thawed. Evaluation studies of UATs are often performed on frozen urine samples, since pneumococcal and *Legionella* polysaccharides are considered to be temperature-stable molecules for moderate time periods [10, 20, 21].

However, the impact of long-term freezing may influence EIA results negatively [22]. When we investigated a 15-year-old urine sample (not included in the study) collected from a patient positive for *L. pneumophila* by culture and PCR, the Binax *L. pneumophila* serogroup 1 EIA was negative, with a mean absorbance ratio of 1.8 in comparison to the negative control (positive reference ratio, >3.0). Low antigen concentration or long-term freezing may have affected the result negatively but was not further investigated. In addition, the ImmuView test was negative but the BinaxNOW *L. pneumoniae* test was faintly positive when the UATs were applied to the sample. This discrepancy of test results may be due to a higher sensitivity of the BinaxNOW test or a false-positive result on a non-serogroup 1 sample, but it has been recommended previously that very weak positive lines should be interpreted cautiously [19]. In our study, we believe that the comparison between the tests was not negatively affected by freezing of the samples, since all samples were frozen for a moderate time period and were tested simultaneously, but we recommend further studies on unfrozen samples.

In conclusion, the sensitivity and specificity of the ImmuView test and the BinaxNOW tests were similar when evaluated on urine samples from infected patients with identified etiology. No difference in specificities between the tests was observed in bacteremic patients with non-pneumococcal non-*Legionella* etiology.
